# Surgical wound infection following open humeral fracture caused by *Mycobacterium houstonense*: a case report

**DOI:** 10.1186/s12879-019-3979-2

**Published:** 2019-04-23

**Authors:** Lei Tian, Zhen Zhang, Zhongju Chen, Ziyong Sun

**Affiliations:** 1 Department of Clinical Laboratory, Tongji Hospital, Tongji Medical College, Huazhong University of Science and Technology, Wuhan, Hubei Province China; 2 Department of Pharmacy, Tongji Hospital, Tongji Medical College, Huazhong University of Science and Technology, Wuhan, Hubei Province China

**Keywords:** *Mycobacteria houstonense*, *Mycobacterium fortuitum group*, TREK diagnostic systems, Microbroth dilution method, *Escherichia coli*

## Abstract

**Background:**

Historically *Mycobacterium houstonense* belongs to the unnamed third biovariant complex of the *Mycobacterium fortuitum* group, which are sorbitol positive. To date, there have been few reports of human infection induced by *M. houstonense* worldwide.

**Case presentation:**

We describe the case of a 68-year-old man with surgical wound infection, following an open humeral fracture, caused by *M. houstonense* and *Escherichia coli.* An implant bone plate had been embedded for internal fixation during surgery on the humeral fracture previously. A week later *E. coli* was isolated from the skin wound secretions. Cefoperazone-sulbactam was used for treatment for two weeks but the infection was not controlled, with a subsequent risk of deep wound infection. External fixation of the fracture was then performed instead of internal fixation. Ten days later, *M. houstonense* was isolated from new wound secretions. *M. houstonense* was identified by the molecular sequencing method. The TREK Diagnostic System was used to test the susceptibility to antibiotics by the microbroth dilution method. Levofloxacin and amikacin were used for treatment according to the results of the susceptibility test and the patient’s condition obviously improved.

**Conclusion:**

To the best of our knowledge, this is the first case in China of human surgical wound infection caused by *M. houstonense* following open humeral fracture. The combination of levofloxacin and amikacin was effective in the treatment of *M. houstonense* infection.

## Background

Rapidly growing mycobacteria (RGM) can produce colonies that do not appear or slowly appear with pigmentation on various solid media within 7 days (most within 3 to 4 days), and contain mycobacterial acid and maintain amylase activity for 3 days or 2 weeks [1, 2]. Traditional classification methods relying on biochemical and phenotypic identification are labor intensive. However, new technologies such as high-performance liquid chromatography, 16S rRNA gene sequencing and PCR restriction fragment length polymorphism analysis have been more recently adopted [1]. The species of RGM capable of producing disease in humans consist primarily of the *Mycobacterium fortuitum* group, the *Mycobacterium chelonae/abscessus* group and the *Mycobacterium smegmatis* group [1]. Historically, the *M. fortuitum* group comprised *M. fortuitum* (formerly *M. fortuitum* biovar *fortuitum*), *Mycobacterium peregrinum* (formerly *M. fortuitum* biovar *peregrinum*) and the taxon known as the unnamed third biovariant complex. The *M. fortuitum* third biovariant complex mainly included sorbitol positive and sorbitol negative organisms, represented by *Mycobacterium houstonense* and *Mycobacterium bonickei*, respectively.

At present, based on the evolution of 16S ribosomal gene (rDNA) sequencing, the unnamed third biovariant complex includes the *Mycobacterium* species: *M. senegalense*, *M. mageritense*, *M. septicum, M. porcinum, M. neworleanense, M. brisbanense, M. houstonense* and *M. bonickei* [1, 3, 4]. Among the third biovariant complex, their 16S rDNA sequences generally differ by 15 bp or fewer [1].

*M. houstonense* was first reported by the CDC (USA) and was isolated from the face wound of a patient who lived in Houston, Texas, so was named *Mycobacterium houston* [4, 5]. To date, almost all of the reports of the now-called *M. houstonense* have been in the USA and Australia [1]. Here we report an infectious case of *M. houstonense* in China. To the best of our knowledge this is the first report of *M. houstonense* in China.

## Case presentation

**Fig. 1:**
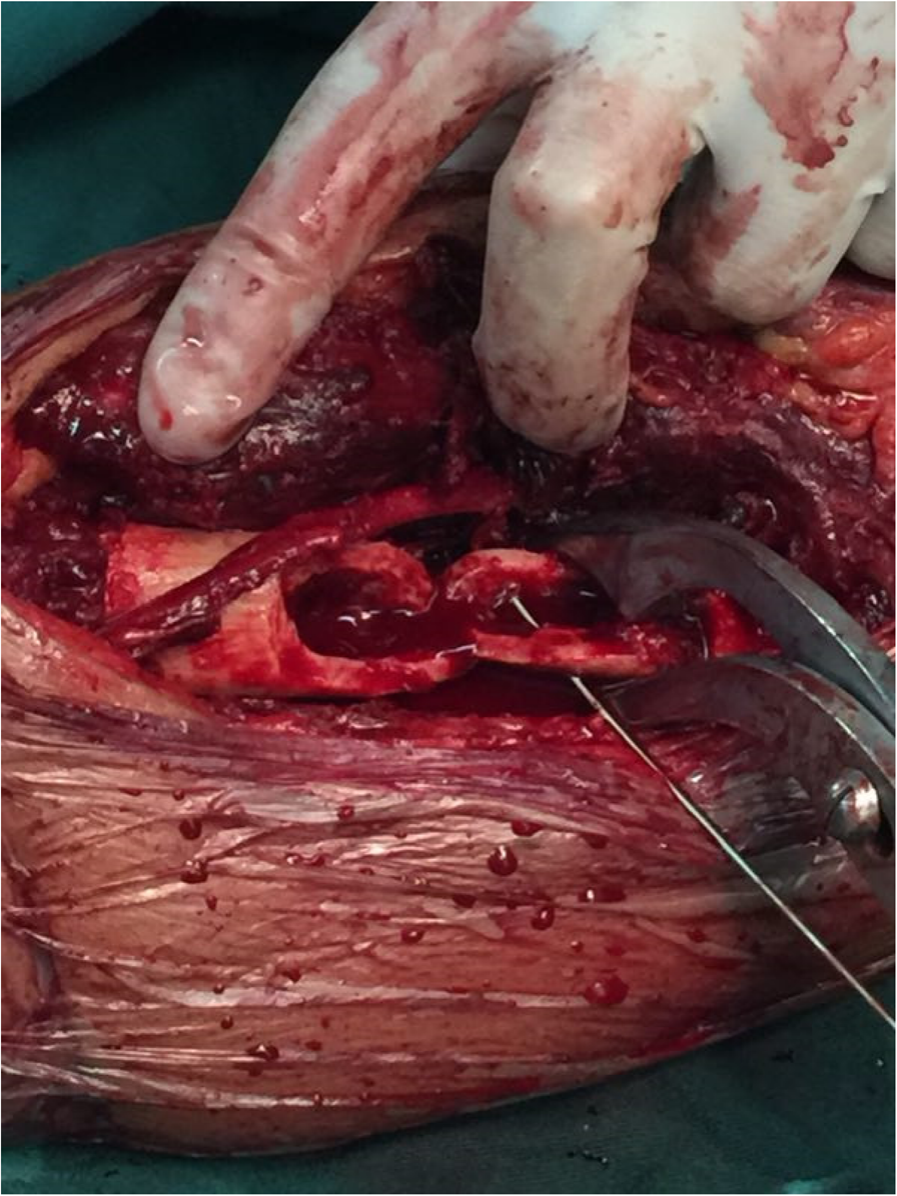
Large bone defects in the middle and lower part of the humerus

**Fig. 2:**
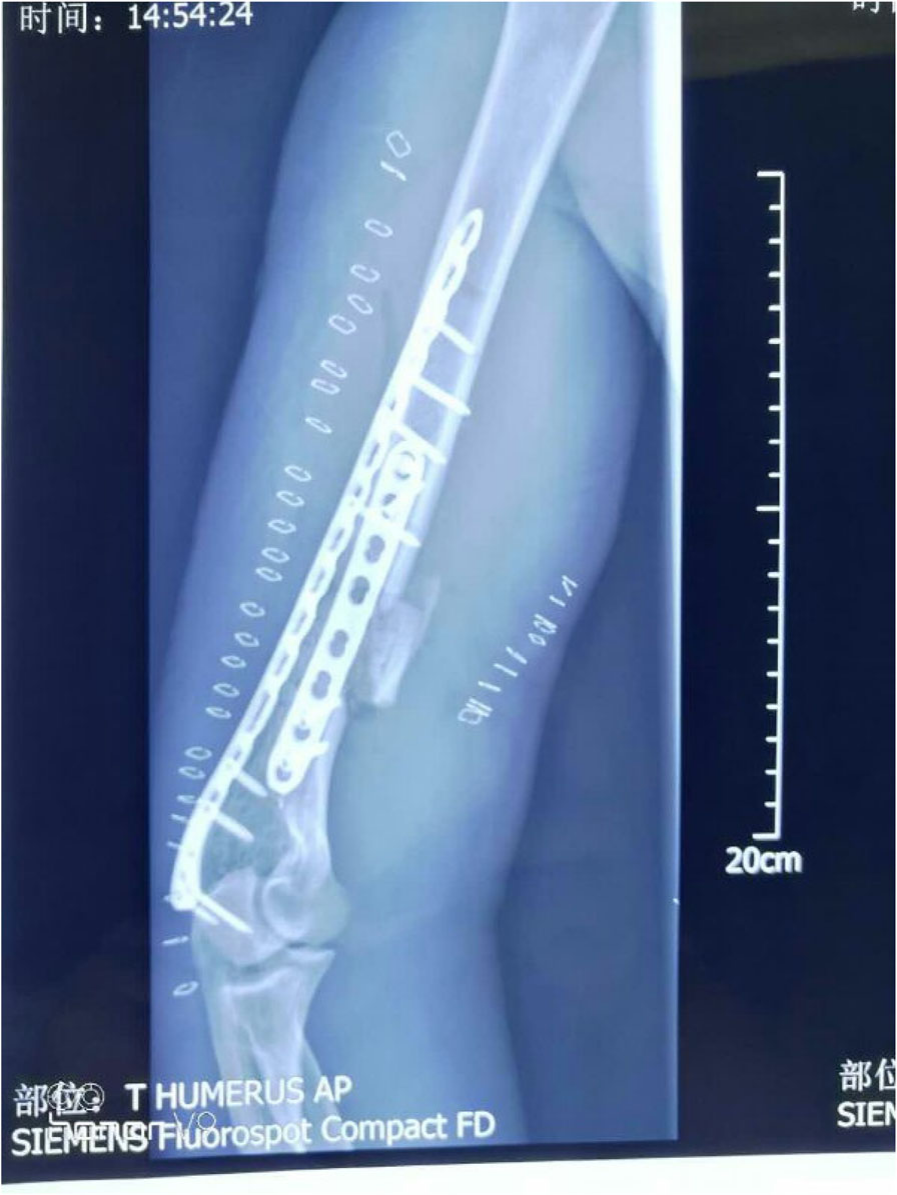
Implant bone plate was embedded for internal fixation

A 68-year-old man had an open fracture of the right humerus due to a fall. The patient was sent to the hospital as an emergency case. The doctor performed debridement and suture of the patient’s wound. When all of the test indexes were normal, surgery of the humeral fracture was undertaken. Large bone defects in the middle and lower parts of the humerus were found during the operation. (Fig. 1) After proper shortening of the fracture end, a bone plate implant was embedded for internal fixation. (Fig. 2).

A week later, wound secretions exuded through the original drainage tube. *Escherichia coli* was isolated from the wound secretion by culture. *E. coli* isolates were multidrug resistant as determined by antimicrobial susceptibility testing using the disk diffusion test. The procedure and interpretation of the results of the antimicrobial susceptibility tests were conducted in accordance with the CLSI 2018 guidelines [6]. Antimicrobial drugs and Mueller–Hinton media for the disk diffusion test were obtained from Oxoid Company, UK. The results showed that the strain was resistant to cefazolin, cefotaxime, cefepime, aztreonam, ampicillin, piperacillin, ciprofloxacin, levofloxacin, moxifloxacin, chloramphenicol, tetracycline and trimethoprim/sulfamethoxazole, but sensitive to gentamicin, amikacin, imipenem, meropenem, ceftazidime, amoxicillin/clavulanate, piperacillin/tazobactam, cefoperazone/sulbactam and cefoxitin. Negative pressure attraction was performed with a progressive artificial skin cover and cefoperazone/sulbactam was used for treatment. Cefoperazone/sulbactam, which combined cefoperazone (2000 mg) with sulbactam (1000 mg), was used via intravenous infusion, once every 12 h.

**Fig. 3:**
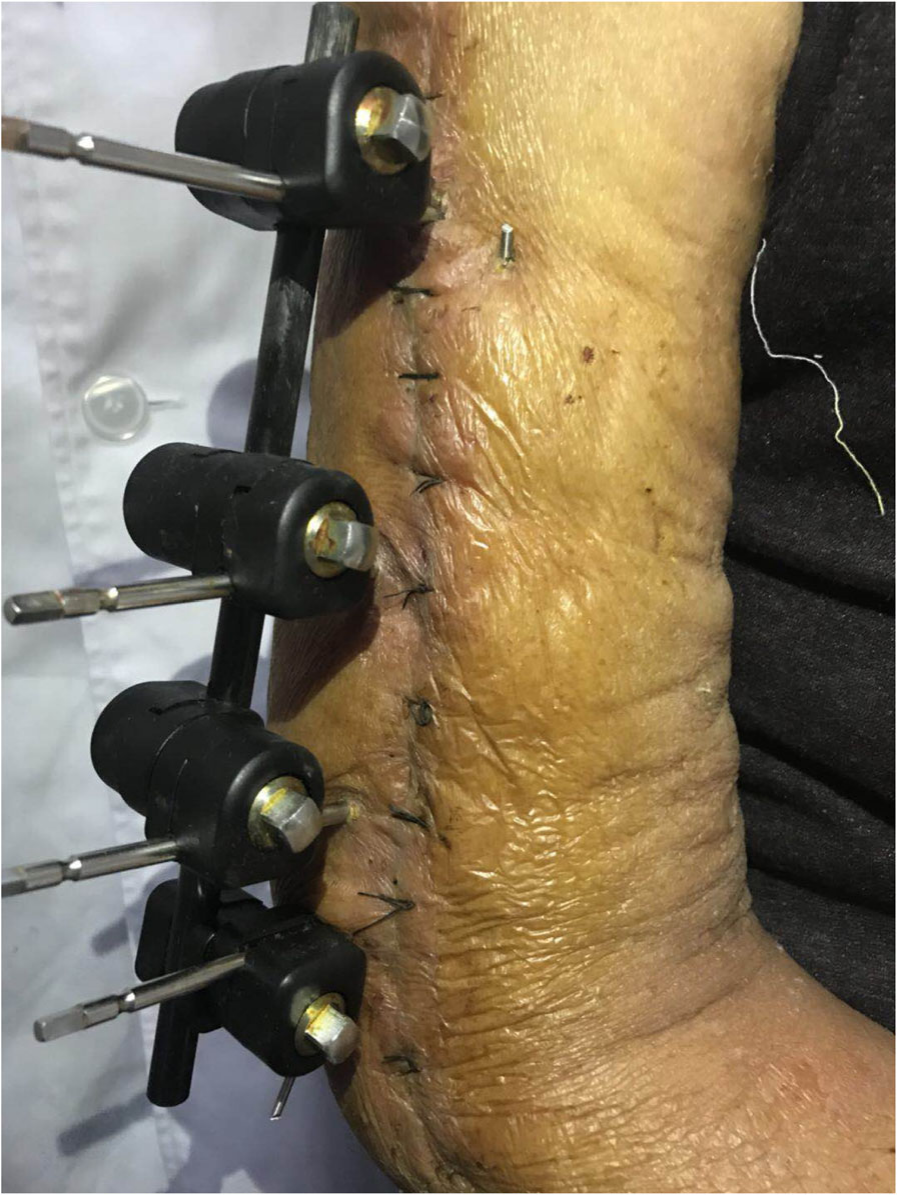
External fixation of fracture were done

Two weeks later, the drainage tube had been closed but yellowish cloudy secretions exuded on the lateral side of the arm incision. The doctors suspected that the deep wound was infected, therefore, re-debridement of the patient’s wound and external fixation of the fracture were performed (Fig. 3).

**Fig. 4:**
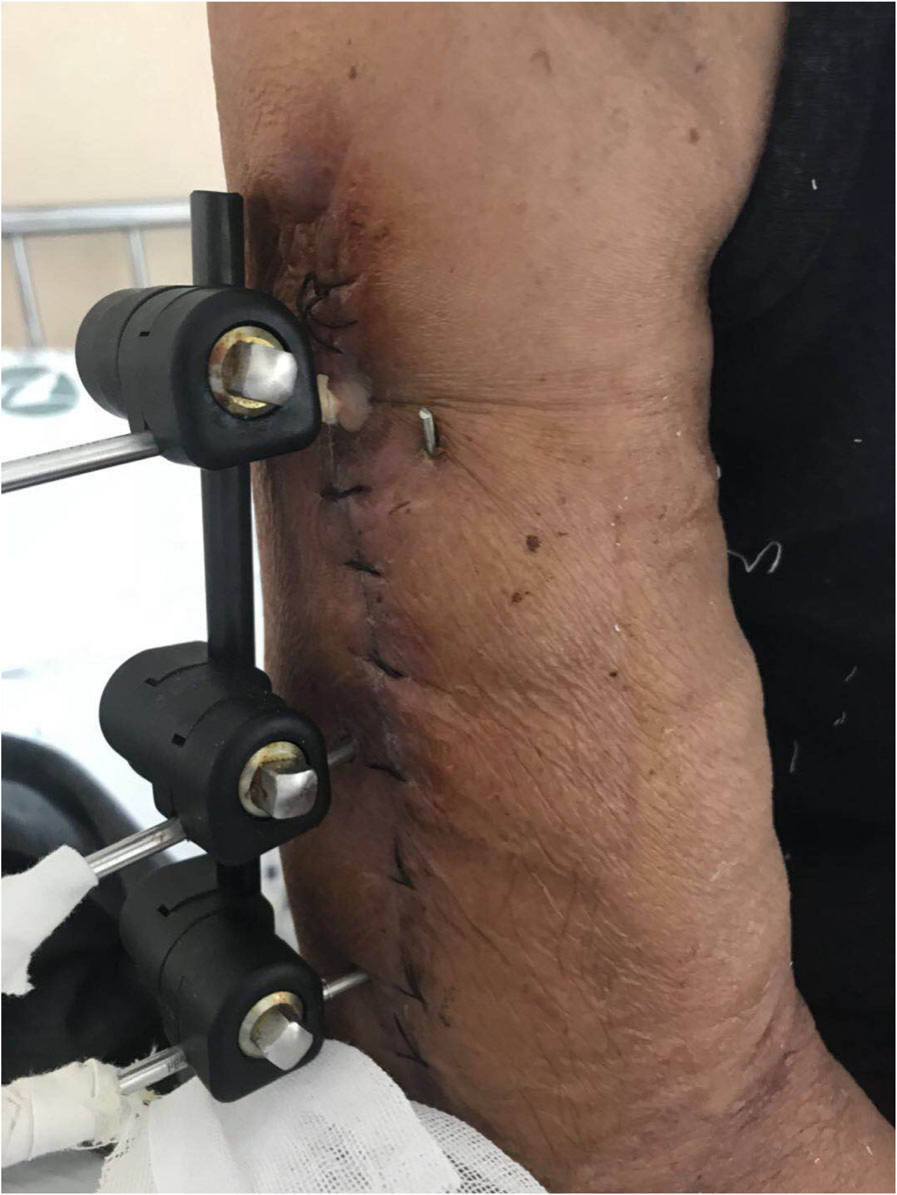
Secretion was seen in the wound

Ten days later, another secretion from the wound was observed (Fig. 4). RGM were isolated from the secretion by culture and were identified as belonging to the *M. fortuitum* group using an IVD-MALDIBIOTYPER (Bruker, Karlsruhe, Germany). The isolated strain was identified as *M. houstonense* by sequencing analysis. Monoclonal colonies were scraped and genomic DNA of the isolate was extracted using a commercial kit (DNeasy Blood and Tissue Kit; Qiagen, Germany). Primer design was based on the reports of Lane(1991) and CLSI MM18-A, and the primers for 16S rRNA PCR were as follows 27F: AGAGTTTGATMTGGCTCAG, 1492R: TACGGYTACCTTGTTACGACTT. The amplification conditions for PCR were based on those of previous reports [7, 8], and a PCR cycler (PTC220, Bio-Rad, USA) and first generation sequencer (Life Technology 2500 DX, ABI, Japan) were used. The amplified products were determined by comparing their restriction patterns with those available in the National Center for Biotechnology Information GenBank database. The results revealed sequence similarity (above 98.58%) with *M. houstonense* (GenBank accession no. NR_042913.1).

The TREK Diagnostic System (Thermo, Germany) was used to test the antimicrobial drug susceptibility of *M. houstonense* by the microbroth dilution method. The antimicrobial drug sensitivity results were interpreted according to the CLSI M24 A2 guidelines [9]. The results, detailed in Table 1, showed that *M. houstonense* was only sensitive to levofloxacin, moxifloxacin and amikacin.

**Table 1:** Antimicrobial sensitivity of *Mycobacterium houstonense*

Antibacterial drugs	MIC (ug/ml)	Breakpoints(μg/mL)			Results
		Susceptible	Intermediate	Resistant	
trimethoprim sulfamethoxazole	4/76	≤2/38	–	≥4/76	resistant
ciprofloxacin	1	≤1	2	≥4	sensitive
moxifloxacin	≤0.25	≤1	2	≥4	sensitive
cefoxitin	≥128	≤16	32–64	≥128	resistant
amikacin	4	≤16	32	≥64	sensitive
doxycycline	≥16	≤1	2–4	≥8	resistant
tegafycline	0.12	–	–	–	–
linezolid	32	≤8	16	≥32	resistant
imipenem	32	≤4	8–16	≥32	resistant
cefepime	≥32	–	–	–	–
amoxicillin-clavulanate	32/16	–	–	–	–
cefatriaxone	≥64	–	–	–	–
minocycline	4	–	–	–	–
tobramycin	8	≤2	4	≥8	resistant
clarithromycin	≥16	≤2	4	≥8	resistant

Note: - No Breakpoint in CLSI M24-A2

A daily intravenous drip of 0.3 g of levofloxacin and injection of 100 ml of sodium chloride, and injection of 0.2 g of amikacin and 250 ml of sodium chloride twice a day, were used for treatment. Three weeks later, the wound was healing well and no secretions were detected.

## Discussion

To the best of our knowledge this is the first report of *M. houstonense* in China. Reports of infection induced by *M. houstonense* are scarce throughout the world. The mechanism of infection with this organism remains unclear. Some studies have reported the isolation of *M. houstonense* from freshwater fish and fish products, especially retail frozen fish [10]. Freshwater fish might therefore be a reservoir of *M. houstonense*. However, in this case, the patient had not recently been exposed to fish products. In the first report of *M. houstonense* in the USA, *M. houstonense* was isolated from a wound in the patient’s face [4, 5]. Similar to many other RGM, *M. houstonense* often inhabits water, soil and dust. Healthcare-associated outbreaks and community-acquired diseases of RGM are usually related to the pollution of water or soil [1]. Stepping on a nail, motor vehicle accidents and compound fractures are typical of the clinical histories seen in patients with RGM infections [11]. In this case, the patient became infected with *M. houstonense* after an open fracture.

RGM are widely distributed and human infections due to RGM have been reported in most geographic areas of the world [1], with the exception of *M. senegalense* that was originally found in Africa and has never been described elsewhere [12]. According to the limited literature, human infections induced by *M. houstonense* have mostly been reported in the USA and Australia to date. This is the first study to report the existence of *M. houstonense* in China, widening the regional distribution of *M. houstonense* throughout the world.

Many experimental methods have been reported for drug susceptibility tests of RGM, such as agar disk elution, the E-test, broth microdilution and agar disk diffusion. Each method has its own advantages and disadvantages. The microbroth dilution method is recommended by the CLSI as a RGM drug sensitivity test, and is currently considered the gold standard. In the CLSI M24 A2 guidelines, the break points of the MIC method for amikacin, tobramycin, cefoxitin, clarithromycin, ciprofloxacin, moxifloxacin, doxycycline, imipenem, meropenem, linezolid and trimethoprim/sulfamethoxazole were specified [9]. In this study, a drug susceptibility test strip of the TREK Diagnostic System (Thermo Company) was used. Besides the 11 drugs mentioned above, cefepime, amoxicillin-clavulanate, cefatriaxone and minocycline were also tested, but no break points were available for the interpretation of these four drugs.

Little is known about potential antimicrobial therapies to treat *M. houstonense* infections. For the treatment of RGM, therapeutic regimens frequently vary depending on the nature of the disease. Single drug therapy for small or localized lesions is usually effective. However, in the case of diffuse or pulmonary infections, it is necessary to combine oral and injectable antibiotics [1]. A report analyzing the *M. fortuitum* group showed > 90% susceptibility or intermediate susceptibility to amikacin, cefoxitin, ciprofloxacin, gatifloxacin, imipenem, levofloxacin, linezolid, sulfamethoxazole or trimethoprim-sulfamethoxazole, and < 90% susceptibility or intermediate susceptibility to clarithromycin, doxycycline and vancomycin [1]. As previously reported, *M. houstonense* isolate 14,133 appeared susceptible to clarithromycin (MIC < 4 μg/ml), but overnight incubation in clarithromycin (0.1 μg/ml) increased the clarithromycin MIC to > 128 μg/ml [1, 13]. The clarithromycin MIC remained at ≤2 μg/ml for organisms incubated in the absence of macrolides [1, 13]. These results indicated that *erm* genes are inducible at the molecular level. Therefore, it is necessary to be careful when interpreting the drug sensitivity of macrolides. *M. houstonense* is known to express the intrinsic macrolide *erm* gene, so inducible drug resistance may occur affecting the macrolide susceptibility results [5, 13]. Our drug susceptibility test showed that *M. houstonense* was only susceptible to levofloxacin, moxifloxacin and amikacin. In this case, the patient was treated with a combination of levofloxacin and amikacin intravenous drip according to the drug sensitivity results. The therapeutic effect was good. However, a standardized treatment for *M. houstonense* remains to be proposed.

A slight wound infection induced by RGM can heal itself and minor wound infections can be resolved be surgical debridement [1]. Many cases of extrapulmonary infections have been reported long before they were cured by surgical debridement alone, but these cases are prone to recurrence within 4–6 weeks [1, 14]. Surgical debridement combined with appropriate antimicrobial therapy can effectively avoid the recurrence of infection [11, 14–16]. In this case, surgical debridement combined with antimicrobial therapy based on the results of drug susceptibility tests was effective in the treatment of *M. houstonense.*

## Conclusion

This case report describes an open fracture infection caused by combined infection of *E. coli* and *M. houstonense*. As the first report of infection caused by *M. houstonense* in China, it aids our understanding of the distribution of *M. houstonense* throughout the world.

## Abbreviation

RGM: Rapidly growing mycobacreria
